# Supplementary material for: Historical visit attendance as predictor of treatment interruption in South African HIV patients: Extension of a validated machine learning model
Source: PLOS Glob Public Health. 2023 Jul 19;3(7):e0002105. doi: 10.1371/journal.pgph.0002105 (PMC10355459; doi:10.1371/journal.pgph.0002105)
Supplement: S1 Table — (DOCX) [file pgph.0002105.s001.docx]

**S1 Table:** Log binomial regression results risk factors for LTFU in a cohort of 191,162 patients initiating antiretroviral therapy in South Africa from Jan 2017-March 2022

**
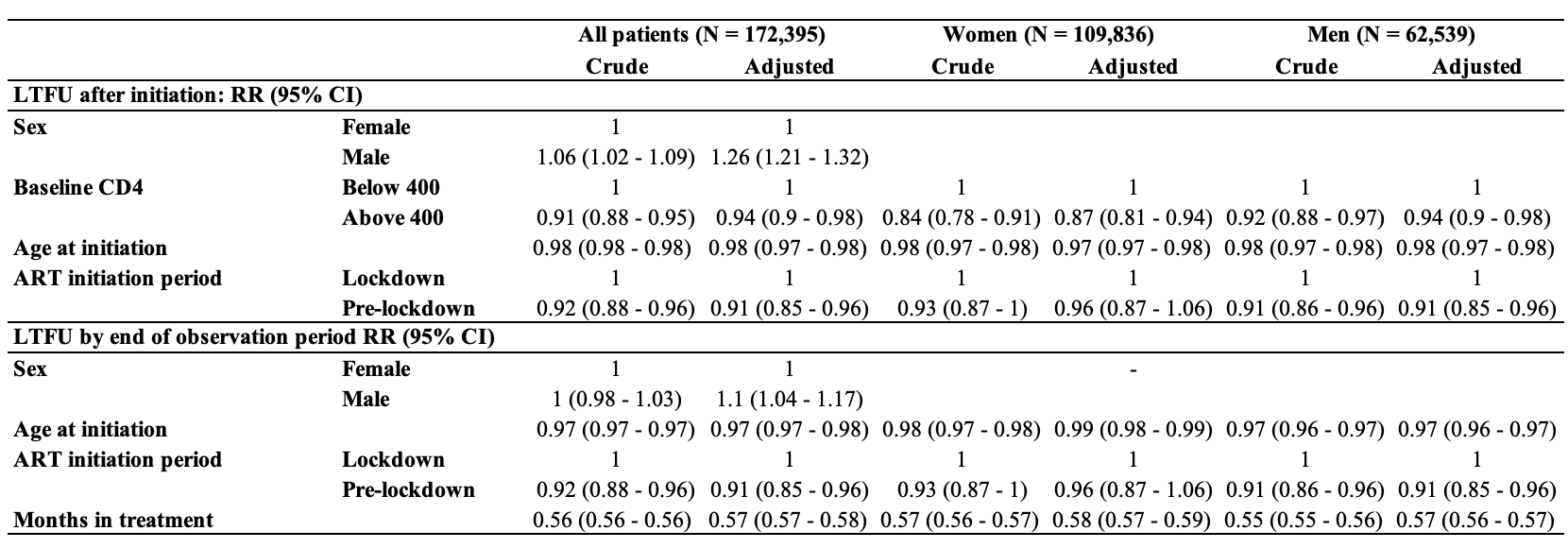
**
