# Supplementary material for: Historical visit attendance as predictor of treatment interruption in South African HIV patients: Extension of a validated machine learning model
Source: PLOS Glob Public Health. 2023 Jul 19;3(7):e0002105. doi: 10.1371/journal.pgph.0002105 (PMC10355459; doi:10.1371/journal.pgph.0002105)
Supplement: Attachment — Submitted filename: plos_ltfu_response_to_reviewers.docx [file pgph.0002105.s003.docx]

**Response to Reviewer comments: Historical visit attendance as predictor of treatment interruption in South African HIV patients: extension of a validated machine learning model**

Dear Editor,

We are grateful to the reviewers for the feedback that has improved the quality and presentation of this paper. We have provided detailed responses to each comment in the document below and have referenced line numbers in the revised manuscript where changes have been made.

Kind regards,

Rachel Esra

***Reviewer #1:***

*Overall comments:*

*I think this is an interesting manuscript to better understand the relationship between sociodemographic, clinical, and care history variables in patterns and prediction of retention. The authors use sophisticated methods (e.g., machine learning) and present interesting analyses to help unpack which variables are most important for understanding future risk of LTFU. I think this is an incredibly important question as to date most attention on risk prediction has focused primarily on sociodemographic and clinical variables rather than observed behavior like visit history. My main suggestions for this article are to better clarify the questions they are trying to answer with each analysis and why it is important. Currently, these are interesting analyses but the overall message, learning points are a bit obscured. I also have additional suggestions for potential analyses that may tighten the message and also to help with clarity.*

*Specific comments:*

*1. Intro*

*• I think the intro is generally well-written and touches on the important points, except I found the second paragraph and bit winding…it is more about how we target retention interventions rather than if they are effective or not.*

**Author Response:**

Thank you for this helpful feedback. The focus of the paper is on identifying those at the highest risk of LTFU and not on the effectiveness of LTFU interventions. To this point, this paragraph gives context for the current demographic subgroups that are known to be at high risk for LTFU – a definition that we are aiming to expand in this paper.

We feel that the current reference to the effectiveness of LTFU interventions is appropriate for the scope of the introduction and have expanded on this topic in the discussion as per the reviewer’s recommendation:

Lines 372 – 384:

“While there is limited quantitative evidence detailing the effectiveness of individual retention interventions in South Africa [5,6], modelling studies have demonstrated improving ART retention is cost saving even at low levels of effectiveness relative to alternatives for HIV spending [32]. A recent systematic review found that ART retention in South Africa was similar in standard care compared to 37 direct-service-delivery treatment interventions including those were facility-based individual models, out-of-facility-based individual models, client-led groups, and healthcare worker-led groups [5]. The direct effectiveness of retention interventions are difficult to quantify given that they are often implemented as part of multifaceted HIV service provision and retrospectively evaluated in an observational study framework [6]. While our work provides some visibility into ART patients who are at a high risk of LTFU, more work needs to be done in evaluating how the risk cohorts defined here can be effectively matched with appropriate retention or directed service delivery treatment modalities.”

*2. I think points that could be highlighted more clearly about how we target. A lot of attention is on just using clinical and sociodemographic characteristics that are not that predictive, but are easily identified. Statistical approaches that include more variables may be a bit better, but are harder to implement (and machine learning may be even a little bit better). But it seems to me that the most important aspect is what kind of data/variables we focus on and put into our models. This is discussed well in the discussion, but I think the main point is that we need to incorporate patients’ observed behaviors (i.e., their care history) into how we do risk assessments. Whether simple variables of care history (prior LTFU, prior lateness) suffice or more advanced machine learning algorithms are needed is I think an important point (and interesting question). I think some highlighting of both the types of data we use and also how sophisticated the algorithm should be discussed.*

**Author Response**

We agree that the complexity of the model used vs. data availability is a valid point of discussion but feel that it is out of the scope of this paper that is focusing on machine learning approaches for routinely available data such as ART patient registry accessed in this data set. We do highlight how the model may be improved by inclusion of socio-behavioural data and highlight this as a current limitation that we would like to address in the discussion:

Lines 414 - 424:

“ART discontinuation in South Africa is mediated by a complex mix of socio-behavioural factors including mobility, stigma and health facility access [34, 35]. A study of HIV patients in the United States demonstrated that data extracted from clinical records, patient mental health evaluations and insurance claims can be leveraged by machine learning methods to produce high precision predictions of patient behaviour across the HIV care cascade [36]. Without socio-behavioural information linked to routine HIV management systems, we use lateness as a signal for the occurrence events that increase the risk of IIT and LTFU. Using only longitudinal visits attendance and baseline clinical outcomes, we are able to predict two thirds of next missed visits. The incorporation of socio-behavioural data could improve the ability of this approach to inform retention interventions to prevent those at risk of disengaging from HIV care.”

*3. For the last paragraph, I wasn’t totally clear on the purpose of the paper. Is it to identify variables that may improve a machine learning algorithm. Is it to identify which variables are most important (sociodemographics, clinical, care history).*

**Author Response**

Thank you for highlighting this. We have restructured the final paragraph of the introduction to give a clearer description of the study objectives and how these relate to our previous work.

Lines 83 - 95:

“In our previous work, we have identified 13 predictors for ART treatment discontinuation relating to age, baseline clinical characteristics and patterns of visits attendance from routinely collected ART patient records [17]. In this study we aim to investigate the causal relationship between these predictors and evaluate the ability of longitudinal patterns in visit attendance to predict future interruptions in ART treatment. This was done by comparing the usage of the same predictors in machine learning methods optimised for predictive power to traditional statistical methods more amenable to causal inference [15,17]. We hypothesize that evaluating non-linear machine learning model predictors in linear statistical models can improve the explainability of high precision predictions. To test this, we first identify baseline and time varying predictors of ART treatment disengagement using traditional statistical methods. We then evaluate the impact of including these predictors into a previously validated machine learning model for ART patient visit attendance [17] and investigate the relationship between these risk factors and machine learning model predictions.”

*4. I would also distinguish between sociodemographic, clinical, care history predictors. I think many people just think of sociodemographic and clinical being important, but what this paper and the authors’ prior work really demonstrates is that care history is likely most important.*

**Author Response**

Yes, this is the central finding of the paper and have addressed it in the opening paragraph of the discussion:

Lines: 331 – 337:

“ART patient retention is a priority for achieving epidemic control in the South African HIV epidemic. To design effective intervention strategies, there is a need for more precise descriptions of the longitudinal changes in the risk of LTFU as well as the characteristics of those who disengage from treatment [24]. Using routinely collected EMR data, we have demonstrated that historical visit attendance is a stronger predictor of the risk of LTFU and IIT compared to demographic and clinical risk factors. Historical visit attendance is highly informative in a machine learning model predicting up to two thirds of next missed ART visits.”

*Methods:*

**Author Response**

We agree with the reviewer that Table 1 with the archetypes and definitions in results leads to difficulty in following in methods section. We have revised the definitions for ease understanding in all methods, tables and figures. We have moved Table 1 (defining archetypes) to the methods sections, reworded the methods and have added additional notations to Figure 1 and the legend for Figure 1 to make these distinctions clearer.

To respond to the individual suggestions by the reviewer on this issue:

*5.I had a bit of hard time keeping track of the definitions and how the analysis was being down. From my understanding, all analyses were done at the level of a scheduled appointment (whether they attended the visit or not). And then each visit (whether attended or not) were classified as on time, late, or IIT depending on when they eventually showed up (and then further categorized based on prior histories). And clarify at what level the analyses are being done.*

For clarification on the level at which definition and analysis was applied, methods were rewritten as follows:

Lines 130 - 152:

**“Operational definition of outcomes**

For the purpose of this study, we considered different operational definitions of treatment interruption on the individual patient level. We assessed longitudinal treatment attendance on a visit by visit basis, by classifying each visit in a patient’s visit trajectory as an interruption in treatment (IIT) if the visit was attended more than 28 days after the scheduled visit date [15,17,20]. On a patient level, we investigated the relationship between the longitudinal pattern of visit attendance and a final outcome of patient retention, where patients were considered LTFU if they were 90 days or more late for a scheduled visit at the end of our observation period in accordance with the South African Department of Health guidelines [19].

**Visit attendance archetypes**

In our previous work, variables describing historical visit attendance including the ratio of visits attended late vs. visits attended on time and the number of historical IITs, were shown to be important in predicting next missed visits [17]. Based on these results and input from the clinical and program teams at The Aurum Health Institute, we developed mutually exclusive and collectively exhaustive visit attendance archetypes that describe historical visit attendance in a single categorical variable (Figure 1). For each visit attended, we defined visits attended within 14 days of a scheduled appointment to be on time and visits attended between 14-28 days of a scheduled appointment to be late (Figure 1). Using these definitions of visits attended on time, visits attended late and IITs, we defined visits archetypes as illustrated and described in Figure 1 and Table 1.”

Table 1 (displayed below) was moved up to the “Methods: Visit archetypes” section for ease of understanding.

*6. Figure 1 may need a more descriptive legend. To me I think the visit in gray is the one being categorized and the red, orange, green represent what has happened in the past (or perhaps it is the rightmost colored dot). A figure like this is great, but I think needs to be a lot clearer.*

We added additional notation and expanded the legend for Figure 1 to add more clarity on how the visits were categorised:

**Fig 1:** Visit archetypes based on longitudinal patterns of ART visit attendance as described in Table 1. Archetypes are mutually exclusive, completely exhaustive and defined by the historical pattern of visit attendance of interruptions in treatment (red), late attendance (orange) and visits attended on time (green). Analysis is focused on how these historical patterns are able to predict attendance at the next visit in the time series (grey).

*7. How can some one return to treatment on time or late? If they have an appointment and are 28 days late they have an interruption, but they may come back at any time after 28 days late and reengage in care. Either way they are more than 28 days late for their last appointment. Looking more in depth at Table 1, the description also makes it sound like this is the visit after someone returns (not their return visit)…but then not sure how their return visit get classified (IIT?).*

**Author Response**

We thank the reviewer for pointing out the inconsistency in this definition. We note that our previous definition of IIT as “a visit not attended within 28 days of the scheduled visits date” was confusing as it implies that a visit does not occur. We have changed this definition to “a visit that was attended more than 28 days after the scheduled visit date”. We believe that this revision alongside the revisions made above, should clarify archetypes described. With this definition, the archetype of patients returning to treatment late and returning to treatment on time should be clearer in defining the type of visit attendance that occurs **after** a visit has been attended more than 28 days from the scheduled visit date.

For clarity we have changed the following definitions throughout the manuscript:

- **Interruption in Treatment (ITT):** a visit that was attended more than 28 days after the scheduled visit date
- **Returning defaulter:**
  - **Visit after ITT on time:** Visit on time where previous visit was an IIT
  - **Visit after IT late:** Visit on time where previous visit was an IIT

*8. For Cox PH model, would clarify what time zero is and also event. Is this time to first IIT or LTFU. Or this a recurrent event analysis looking forward from each made visit.*

**Author Response**

This is a recurrent event analysis looking forward from each visit made with each IIT recorded as an event. This has been clarified in the methods:

Line 200:

“In this recurrent event analysis, each IIT experienced was recorded as an event and patients who did not experience an IIT were censored”

*9.How do the original 13 variables different from visit archetypes. My understanding is that previous history was already included in the original machine learning model (and those models may more flexibly characterize complex trajectories over time)…I do understand more after looking at Table 2 but would be nice to understand in methods so questsion becomes clear.*

*10. In general, would try to be specific about the question each analysis is trying to answer. My sense is that you are trying to see whether visit archetypes are good enough and then don’t even require machine learning (or something to that end)…but I am not sure. Similar to machine learning vs. Cox PH*

**Author Response**

We agree that the specific analytic aims were unclear in the original version of this manuscript. We have clarified these aims in the last paragraph of the introduction, methods and in the presentation of the results.

Based on the previous feedback we have clarified this in the last paragraph of the introduction:

Lines 83 – 95:

“In our previous work, we have identified 13 predictors for ART treatment discontinuation relating to age, baseline clinical characteristics and patterns of visits attendance from routinely collected ART patient records [17]. In this study we aim to investigate the causal relationship between these predictors and evaluate the ability of longitudinal patterns in visit attendance to predict future interruptions in ART treatment. This was done by comparing the usage of these predictors in machine learning methods optimised for predictive power to traditional statistical methods more amenable to causal inference [15,17]. We hypothesize that evaluating non-linear machine learning model predictors in linear statistical models can improve the explainability of high precision predictions. To test this, we first identify baseline and time varying predictors of ART treatment disengagement using traditional statistical methods. We then evaluate the impact of including these predictors into a previously validated machine learning model for ART patient visit attendance [17] and investigate the relationship between these risk factors and machine learning model predictions.”

We have added an additional paragraph into the method section, detailing the specific analytical aims:

Lines 162-170:

**“Data analysis**

As described previously, determining causal relationships between predictors in machine learning algorithms is difficult due to non-linearity and the collinearity [ref]. The aim of this analysis was to compare the explainability and predictive performance of a non-linear machine learning model and linear traditional statistical methods on the same analytical problem. In order to do this we identified linear predictors of treatment disengagement using traditional statistical methods, evaluated the impact of including these predictors in a machine learning model with a validated set of high performing predictors [17] and finally, compared the predicted risk associated with visit archetypes between the two analytical methods.”

*Results
11. Should you also describe characteristics of visits as I think most analyses are being on that level?*

**Author Response**

The characteristics of the visits are detailed in Table 2 (previously included as Table 1).

*12. What does “returned to treatment after initiation” mean. I think it represent someone who came back a second time (i.e., not LTFU the day of initiation) but also sounds a bit like reengagement. May want to rephrase.*

**Author Response**

This is noted and has been reworded as “patients who attended at least one additional visit after initiation”

*13. I don’t fully understand Figure 2 and how the dynamics of LTFU and then return to care play out with the blue and yellow lines (related to prior comment).*

**Author Response**

We believe the revision of the definition of ITT and how a patient may return to treatment once this has occurred has been addressed by our previous revisions. We have reworded the figure legend in Figure 2 to make this clearer and have amended the figure legend for further understandability.

**Figure 2:** Longitudinal ART clinic visit attendance in the first two years of antiretroviral therapy in a cohort of 191,162 patients initiating antiretroviral therapy in South Africa from Jan 2017-March 2022. Purple dots represent the proportion of patients that do not return to treatment after treatment initiation. Green lines represent the monthly proportion of visits that are attended more than 28 days after a scheduled appointment (ITT) and yellow lines represent the proportion of monthly visits attended by patients within 28 days of a scheduled visit date after previously experiencing an ITT (Return after ITT).

*14. Table 2 seems to have some of the most interesting findings from the study. I think would be really interesting to step and consider the important to ask here, and I think that has to do with the types of variables included. I see 3 categories: sociodemographic (age, sex); clinical (VLs), but then more are related to care history (duration in care, appointments, lateness ratio, visit archetypes). Could examine what prediction looks like with or without care history (e.g., how good does sociodemographics do). Could examine using visit archetypes vs. the other nuanced care history variables. Ultimately, I think it comes down to what is most useful. A clinician can easily identify sex, age, and a visit archetype, but can’t really calculate lateness ratios (etc.). Could try and understand what is a minimum feasible to get good prediction.*

**Author Response**

We thank the reviewer for this suggestion and have included this categorisation of predictors in the discussion. As has been discussed in our previous publication [Esra et al. 2023] and in the discussion of this paper, the application of machine learning methods combine predictors using non-linear mathematical relationships which in turn cannot be interpreted as “risk predictors” as such. The purpose of this study is to extend our understanding of those predictors so that we might use these methods to inform programmatic strategy as well as generate high prevision prediction. If demographics were important predictors on their own, they would have been selected by the machine learning models in the variable selection process we have detailed in that publication. Additionally, the results in table S1 from the log binomial regression and survival analysis report the difference between the crude and adjusted analyses, demonstrating how demographics such as age and sex were not shown to predictive of ITT and LTFU relative to historical visit attendance.

*Discussion
15 I think the discussion is good and focuses on the important points, particularly the important of care history. I think a sharper focus on the questions at hand could be helpful (but I think will come from addressing my comments in other sections).*

*16 Additional citations to consider for intro or discussion are PMID 33948789, 33105396, 32173743, 31661487. For disclosure, I am a co-author on these studies so feel free to include or not include based on your judgement. I think they may complement what this study does by discussing how we think about risk of LTFU, its time-varying nature, and what types are data needed to get better understandings, which I think are all very important questions that this paper helps unpack.*

**Author Response**

Thank you for bringing our attention to other relevant work. We have included these in the discussion to support our concept of ART treatment adherence as dynamic temporal process:

Lines 347 - 355:

“When assessing trends in IIT, we observed that 85% of patients who miss a visit by more than 28 days return for a subsequent visit, implying that cross-sectional estimates of LTFU are not a good indicator of current treatment coverage. We observed temporal landmarks in ART visit attendance, with rates of IIT being lowest at the 1-year and 2-year landmarks. This demonstrates that ART retention and treatment engagement are dynamic processes and current approaches that do not consider temporal trends are not appropriate for characterising gaps in care over time [28]. Understanding this is critical in informing cross-sectional estimates of treatment coverage, given the large variation in the sensitivity and specificity of current methods to assess ART treatment adherence [29].”

***Reviewer #2:***

1. *Background
   Additionally, it would be helpful to clarify the specific research questions or hypotheses being addressed in this study. While the overall aim of the study is clear, it may be useful to explicitly state the research questions or hypotheses that were tested using the machine learning and traditional statistical methods.*

**Author Response**

We have restructured the final paragraph of the introduction to give a clearer description of the study objectives and how these relate to our previous work.

Lines 83-95:

“In our previous work, we have identified 13 predictors for ART treatment discontinuation relating to age, baseline clinical characteristics and patterns of visits attendance from routinely collected ART patient records [17]. In this study we aim to investigate the causal relationship between these predictors and evaluate the ability of longitudinal patterns in visit attendance to predict future interruptions in ART treatment. This was done by comparing the usage of the same predictors in machine learning methods optimised for predictive power to traditional statistical methods more amenable to causal inference [15,17]. We hypothesize that evaluating non-linear machine learning model predictors in linear statistical models can improve the explainability of high precision predictions. To test this, we first identify baseline and time varying predictors of ART treatment disengagement using traditional statistical methods. We then evaluate the impact of including these predictors into a previously validated machine learning model for ART patient visit attendance [17] and investigate the relationship between these risk factors and machine learning model predictions.”

*2. Methods*

*The methods section could benefit from a clearer explanation of what "previous visit archetypes" are and how they were used in the analysis.*

**Author Response**

We have revised the manuscript to clarify these definitions with following:

For clarification on the level at which definition and analysis was applied, methods were rewritten as:

Lines 130 - 150:

**“Operational definition of outcomes**

For the purpose of this study, we considered different operational definitions of treatment interruption on the individual patient level. We assessed longitudinal treatment attendance on a visit by visit basis, by classifying each visit in a patient’s visit trajectory as an interruption in treatment (IIT) if the visit was attended more than 28 days after the scheduled visit date [15,17,20]. On a patient level, we investigated the relationship between the longitudinal pattern of visit attendance and a final outcome of patient retention, where patients were considered LTFU if they were 90 days or more late for a scheduled visit at the end of our observation period in accordance with the South African Department of Health guidelines [19].

**Visit attendance archetypes**

In our previous work, variables describing historical visit attendance including the ratio of visits attended late vs. visits attended on time and the number of historical IITs, were shown to be important in predicting next missed visits [17]. Based on these results and input from the clinical and program teams at The Aurum Health Institute, we developed mutually exclusive and collectively exhaustive visit attendance archetypes that describe historical visit attendance in a single categorical variable (Figure 1). For each visit attended, we defined visits attended within 14 days of a scheduled appointment to be on time and visits attended between 14-28 days of a scheduled appointment to be late (Figure 1). Using these definitions of visits attended on time, visits attended late and IITs, we defined visits archetypes as illustrated and described in Figure 1 and Table 1.”

Table 1 (displayed below) was moved up to the “Methods: visit archetypes” section for ease of understanding.

**Table 1:** Visits archetype definitions based on longitudinal patterns of ART visit attendance

We added additional notation and expanded the legend for Figure 1 to add more clarity on how the visits were categorised:

**Fig 1:** Visit archetypes based on longitudinal patterns of ART visit attendance as described in Table 1. Archetypes are mutually exclusive, completely exhaustive and defined by the historical pattern of visit attendance of interruptions in treatment (red), late attendance (orange) and visits attended on time (green). Analysis is focused on how these historical patterns are able to predict attendance at the next visit in the time series (grey).

*3. Overall*

*Overall, the manuscript appears to be well-written and provides valuable insights into patient retention in HIV treatment in South Africa. The use of machine learning to predict patient outcomes based on historical data is particularly innovative and could have important implications for improving patient retention strategies. However, the manuscript could benefit from additional detail on the specific interventions that could be implemented based on the findings. Additionally, further discussion of potential limitations of the study, such as the lack of unique patient identifiers and the potential for misclassification of patient outcomes, could help contextualize the results.*

Authors response:

We thank the reviewer for these encouraging comments and suggestions, we have included a paragraph in the discussion on how current evidence on retention interventions:

Lines 372 - 384:

“While there is little quantitative evidence to support the effectiveness of individual retention interventions in South Africa [5,6], modelling studies have demonstrated improving ART retention is cost saving even at low levels of effectiveness relative to alternatives for HIV spending [32]. A recent systematic review found that ART retention in South Africa was similar in standard care compared to 37 direct-service-delivery treatment interventions including those were facility-based individual models, out-of-facility-based individual models, client-led groups, and healthcare worker-led groups [5]. The effectiveness of retention interventions are difficult to quantify given that they are often implemented as part of multifaceted HIV service provision and retrospectively evaluated in an observational study framework [6]. While our work provides some visibility into ART patients who are at a high risk of LTFU, more work needs to be done in evaluating how the risk cohorts defined here can be effectively matched with appropriate retention or directed service delivery treatment modalities.”

The limitation of missing unique identifiers is also addressed in the discussion:

Lines 404 – 413:

“Due to the absence of unique patient identifiers, we were unable to account for patient mobility or validate outcome reporting. As a result, it is likely that a subset of patients classified as experiencing IIT or becoming LTFU were attending treatment at other facilities. Comparison of facility level outcomes to a South African national laboratory cohort demonstrates that HIV patient retention is underestimated at the facility level where undocumented patient transfers reflect as discontinuations in treatment [3]. Over six years of treatment, retention in care at the national level accounting for patient mobility was 63% relative to 29% at a facility level [3]. We plan on focusing future work on aligning viral load testing records with longitudinal patient attendance records as an improved method of ascertaining and predicting individual level treatment status.”

The limitations and relevance of the model have been discussed in depth on our previous publication on model validation and extension. This is well noted and we have included a reference to this in the discussion around model sensitivity:

Lines 394 - 402:

“The sensitivity of the model to correctly predict IIT is a function of prevalence of the IIT as well as the occurrence of consistent patterns preceding IIT. Due to this, model development towards either sensitivity or precision is context dependant as has been discussed in our previous work [17].”
