# Supplementary material for: Historical visit attendance as predictor of treatment interruption in South African HIV patients: Extension of a validated machine learning model
Source: PLOS Glob Public Health. 2023 Jul 19;3(7):e0002105. doi: 10.1371/journal.pgph.0002105 (PMC10355459; doi:10.1371/journal.pgph.0002105)
Supplement: Attachment — Submitted filename: 090523_response_to_reviewers.docx [file pgph.0002105.s004.docx]

**Response to Reviewer comments: Historical visit attendance as predictor of treatment interruption in South African HIV patients: extension of a validated machine learning model**

Dear Editor,

We are grateful to *Reviewer 1* for the continued feedback and have taken their comments under consideration. We have made substantial changes to the introduction that we believe have improved the clarity of the research aims and implications of the findings of this paper. We have provided detailed responses to each comment in the document below and have referenced line numbers in the revised manuscript where changes have been made.

Kind regards,

Rachel Esra

Reviewer #1: Overall comments:
Thank you for the authors’ for addressing my previous comments. I still had a few points for clarification. I still there is additional clarity around the question at hand and why its important.

*Review Comment:* *I would remove all references to causality. The issue with causal inference isn’t a statistical one due to non-linearity and collinearity. There are more complex issues at hand with time-varying mediation-confounding, etc. and unmeasured confounding that can’t be handled statistically.*

**Author response:** We are in agreement with this and have either removed references to causality from text or replaced the phrase with “explainability” or other clarifying phrases:

Lines 138 – 147:
“Previously, we identified 13 predictors for ART treatment discontinuation relating to age, baseline clinical characteristics and patterns of visits attendance from routinely collected ART patient records [17]. Here, we aim to expand the explainability of these predictors as a means of providing more generalised descriptions of the population at risk of IIT and the underlying drivers of risk.”

Lines 282 - 288:

“As described previously, clearly describing and explaining relationships between predictors in machine learning algorithms is difficult due to non-linearity and the collinearity [17].

Lines 498 - 499:

“Collinearity and the non-linear nature of predictors in our previously validated machine learning model limit explainability of risk factors identified as predictive of IIT [17].”

*Review Comment:* *I agree with the author’s that discussion of the effectiveness of LTFU interventions is relevant, but as a reader, the narrative gets lost. Its not until the end of the intro or beginning of methods that I start to understand what the manuscript is about. Would just consider reframing/organizing so the main narrative stays clear.*

*I appreciate the authors’ perspective that discussion of what variables are included are not within the scope of this manuscript, though I am not sure I agree. Again, this may be partially because I don’t follow the intro narrative for the specific questions and their implications. Machine learning also seems potentially useful when data is complex and would require a lot of decision-making beforehand. Considering these issues also directly guides what types of statistical issues and questions are most important to address.*

**Author response:**

We appreciate the reviewer’s input on the clarity of the manuscript and have restructured the introduction and discussion of this paper to better explain the narrative and specific questions we are addressing:

- In the introduction we have restructured the background framing the issue of what is currently known about risk factors for LTFU in South Africa HIV patients on ART, why these risk factors are difficult to measure and how there is little evidence that interventions targeting populations described by demographic and clinical characteristics improve retention outcomes in this context
- We introduce the concept of historical visit attendance as independent predictor of interruption in ART, including a better description of our previous work and why the individual and relative role of the predictors highlighted in that model are difficult to explain using machine learning methods alone
- We have restructured the final paragraph to better delineate between the two aims of this study to (1) compare the performance of the same set of 13 predictors in machine learning and traditional statistical methods and (2) evaluate the performance of historical visit archetypes on predicting the same outcome in both machine learning and traditional statistical methods.

Lines 100 – 147:

“

While South Africa has the largest HIV treatment programme globally, it is currently estimated that a quarter of the 7.5 million people living with HIV (PLHIV) are not on antiretroviral treatment (ART) [1]. ART is lifelong and stopping treatment results in rapid viral rebound, putting patients at an individual risk for AIDS-defining illness and increasing the risk of viral transmission [2]. Retention on ART remains a challenge in South Africa where 11 - 28% of patients become lost to follow up (LTFU) within the first two years of treatment initiation [3,4].

ART treatment interruption in South Africa is likely mediated by a complex mix of socio-behavioural factors including mobility, stigma and health facility access [5,6]. Cohort studies indicate that the risk of LTFU varies over time [7,8] and many patients iteratively transition in and out of treatment [9], making behavioural drivers of ART retention difficult to define longitudinally. Without socio-behavioural information linked to routine HIV management, many retention interventions are focused on broad demographic sub-populations with perceived elevated rates of LTFU, including men, those diagnosed with HIV at younger ages and those initiating treatment with lower CD4 counts [10, 11]. However, little evidence supports the effectiveness of this approach .[12,13].

Innovative approaches to understanding and addressing risk of disengagement from HIV care are needed. Traditional statistical methods such as regression and survival analysis are frequently used to enumerate factors that describe elevated risk of LTFU [7-11]. Though widely adopted due to their ease of computation and explainability, these methods are limited in terms of accurately modelling collinearity, interaction effects and non-linear relationships between predictors [14] and are thus unable to uncover the complex mechanisms driving risk of disengagement from care. In contrast to this, machine learning methods are able to account for non-linear patterns often present in routinely collected observational data, and are increasingly being used to identify high risk subgroups of populations with suboptimal HIV service utilisation in low- and middle-income contexts [15-18].

We have previously described a machine learning algorithm able to predict up to two thirds of missed ART clinic visits using only visit attendance and routinely collected clinical information [16,17]. In this model, patterns of historical visits attendance ranked higher than baseline demographic and clinical characteristics when predicting next missed visits [17]. While this model is able to predict the risk of disengagement from care at the level of on an individual patient and visit, the approach is still limited in terms of ability to infer relationships between predictors and interpret both the individual and relative role of potential predictors of treatment interruptions [17].

Previously, we identified 13 predictors for ART treatment discontinuation relating to age, baseline clinical characteristics and patterns of visits attendance from routinely collected ART patient records [17]. Here, we aim to expand the explainability of these predictors as a means of providing more generalised descriptions of the population at risk of IIT and the underlying drivers of risk. We assess the relative contribution of historical visit attendance in predicting risk of treatment interruption, by defining mutually exclusive and collectively exhaustive visit attendance archetypes encompassing this information in a single categorical variable. We then evaluate the predictive ability of the archetypes alone and in combination with the previously identified demographic and clinical predictors using both machine learning and traditional statistical methods.

”

We have additionally clarified these points in our description of historical visit archetypes development and in the specific analytical aims in the methods section:

Lines 236 - 269:

**“Visit attendance archetypes**

In our previous work, variables describing historical visit attendance including the ratio of visits attended late vs. visits attended on time and the number of historical IITs, were shown to be more important in predicting next missed visits than baseline demographic and clinical features [17]. Based on these results and input from the clinical and program teams at The Aurum Health Institute, we developed mutually exclusive and collectively exhaustive visit attendance archetypes that describe historical visit attendance in a single categorical variable (Figure 1). For each visit attended, we defined visits attended within 14 days of a scheduled appointment to be on time and visits attended between 14-28 days of a scheduled appointment to be late (Figure 1). Using these definitions of visits attended on time, visits attended late and IITs, we defined visit archetypes as illustrated and described in Figure 1 and Table 1.”

Lines 281 - 288:

“**Data analysis**

As described previously, the explainability of relationships between predictors in machine learning algorithms is difficult due to non-linearity and the collinearity [17]. While our previous work ranked historical visit attendance highly in predicting next missed ART visits, using machine learning methods alone, we are unable to assess the relative contribution of historical visit attendance to other variables included. Here we evaluate the individual and combined predictive ability of historical visits attendance, baseline demographic and clinical characteristics comparing traditional statistical approaches with machine learning methods.”

*Review Comment:* *I still think for Table 3 would be important to consider the conceptually different categories for data types. Looking at the previously published paper, variables on visit history were the most important, sociodemographics less so. Is the questions here, will adding archetypes—when you already included other visit history in the machine learning model—improve prediction? And the rationale for adding archetypes is because they were associated with outcomes using more routine statistical methods (although those models didn’t include visit history). Again I may not be following correctly, but I would not anticipate that would add much. There are many practical considerations to consider to how one would want to add additional variables like visit archetypes to an existing model. I don’t particularly follow the rationale for the approach taken here. Some conceptual distinction between the variables used in the prior models vs these archetypes should be made (outside of the history of how the authors’ have chosen to use them).*

**Author response:** We have adapted the relevant table heading and variable grouping to better clarify the distinction between the various predictors included in the three models compared in this analysis:

**Table 3:** Model performance in analysis of prediction of interruption in treatment in a cohort of 191,162 patients initiating antiretroviral therapy in South Africa from Jan 2017-March 2022. We compared the performance of (A) the top 13 predictors from the validated CatBoost model, (B) the addition of previous visit archetypes to the validated CatBoost model and (C) a model using only previous visit archetypes as predictor.

We believe that the clarification to the introduction and methods section detailed in our previous response have addressed the reviewer’s comment around the specific research aims and choice in analytical methods. These findings and their implications are also further discussed in lines 498-513:

“Collinearity and the non-linear nature of predictors in our previously validated machine learning model limit explainability of risk factors identified as predictive of IIT [17] and therefore the ability to understand drivers of risk at the individual level and intervene accordingly before a treatment interruption occurs. In this study, both machine learning optimised for predicting a binary outcome and traditional statistical methods identified historical visit attendance as a strong predictor of IIT. Lateness and repeat patterns of lateness are strong predictors of IIT, irrespective of current age, age at ART initiation, sex and baseline CD4. This is aligned with results observed in historical cohort studies reporting that the timeliness of clinic attendance is a good predictor of viral load suppression and the development of ART resistance [20,31]. In the absence of observable risk factors, we believe lateness is an actionable behavioural flag for a patient that may become LTFU in future but is currently present at a healthcare access point. This finding may inform patient retention strategies by identifying patients who are good candidates for prioritised interventions - those who are demonstrating a willingness to be on treatment and experiencing some external barrier. This creates the potential for targeted proactive intervention, as opposed to resource intensive retrospective tracing.”
