# Supplementary material for: Historical visit attendance as predictor of treatment interruption in South African HIV patients: Extension of a validated machine learning model
Source: PLOS Glob Public Health. 2023 Jul 19;3(7):e0002105. doi: 10.1371/journal.pgph.0002105 (PMC10355459; doi:10.1371/journal.pgph.0002105)
Supplement: Attachment — Submitted filename: 260523_response_to_reviewers.docx [file pgph.0002105.s005.docx]

**Response to Reviewer comments: Historical visit attendance as predictor of treatment interruption in South African HIV patients: extension of a validated machine learning model**

Dear Editor,

Thank you for your clear and constructive feedback on highlighting the main findings and recommendation of this paper. We have incorporated these recommendations into the abstract, methods and discussion have provided detailed responses to each comment in the document below.

Kind regards,

Rachel Esra

Editor comment: *Please review your reference list to ensure that it is complete and correct. If you have cited papers that have been retracted, please include the rationale for doing so in the manuscript text, or remove these references and replace them with relevant current references. Any changes to the reference list should be mentioned in the rebuttal letter that accompanies your revised manuscript. If you need to cite a retracted article, indicate the article’s retracted status in the References list and also include a citation and full reference for the retraction notice.*

**Authors response:** We apologise for the error as the referencing in our revised manuscript was out of date and had the accidental inclusion of a 37^th^ reference that does not exist. We have adjusted the in-text referencing (as reflected in the tracked changes on our document) to reflect the reference list which remains unchanged from the previous submission. W

*Editor comment*: *There is limited engagement in the text with the contents of Table 3, which is the key evidence in response to the question on whether categorical visit attendance is on its own a valid predictor. The discussion summarizes across the models ("Historical visit attendance is highly informative in a machine learning model predicting up to two thirds of next missed ART visits" - presumably a reference to the sensitivity >60% of the 2 models with clinical and demographic variables also included) and the abstract highlights the performance of the model without categorical adherence. Please provide greater interpretation in the text of the results shown in Table 3 and elaborate in the discussion what the implications are for both variable type and statistical approach recommended for use moving forward.*

*Reviewer 1: Thank you for the authors’ for addressing my previous comments. I think the manuscript is improved and easier to follow with these changes. I do still find some of the explanation dense, particularly related to Table 3, and I feel a general reader who is not familiar with differences between examining causality vs. prediction would struggle. Ultimately, what I believe the authors are saying and an underlying point is that machine learning algorithms are complex and model complex relationships, thus they are not readily useable in clinical settings (unless implemented in an EHR that can do the computations in the real-time in the background). Essentially, they are a black box. Visit archetypes, however, are readily identifiable by clinicians/practitioners—either with EHRs or not—and without any need for understanding complex relationships between variables. Thus, it is useful and important to understand the performance of these archetypes. I think bringing out these practical implications would be helpful in the results and discussion.*

**Authors response:** We appreciate the feedback from both the editor and the reviewer on highlighting the key findings of the paper by expanding on the results on Table 3 and making a clearer recommendation on the implications of these findings. We have responded to both comments below by restructuring the abstract, adding an expanded and clearer interpretation of the findings in Table 3 in the results and expanding and clarifying the implications of these findings in the discussion.

The abstract has been updated as follows (with relevant clarifications and additions underlined here):

“Retention of antiretroviral (ART) patients is a priority for achieving HIV epidemic control in South Africa. While machine-learning methods are being increasingly utilised to identify high risk populations for suboptimal HIV service utilisation, they are limited in terms of explaining relationships between predictors. To further understand these relationships, we implemented machine learning methods optimised for predictive power and traditional statistical methods. We used routinely collected electronic medical record (EMR) data to evaluate longitudinal predictors of lost-to-follow up (LTFU) and temporal interruptions in treatment (IIT) in the first two years of treatment for ART patients in the Gauteng and North West provinces of South Africa. Of the 191,162 ART patients and 1,833,248 visits analysed, 49% experienced at least one IIT and 85% of those returned for a subsequent clinical visit. Patients iteratively transition in and out of treatment indicating that ART retention in South Africa is likely underestimated. Historical visit attendance is shown to be predictive of IIT using machine learning, log binomial regression and survival analyses. Using a previously developed categorical boosting (CatBoost) algorithm, we demonstrate that historical visit attendance alone is able to predict almost half of next missed visits. With the addition of baseline demographic and clinical features, this model is able to predict up to 60% of next missed ART visits with a sensitivity of 61.9% (95% CI: 61.5-62.3%), specificity of 66.5% (95% CI: 66.4-66.7%), and positive predictive value of 19.7% (95% CI: 19.5-19.9%). While the full usage of this model is relevant for settings where infrastructure exists to extract EMR data and run computations in real-time, historical visits attendance alone can be used to identify those at risk of disengaging from HIV care in the absence of other behavioural or observable risk factors.”

The following has been added to the results to expand on the interpretation of the findings in Table 3 (lines 388 – 397):

“When using the original set of 13 predictors, model performance decreased relative to previous iterations [17] when trained and tested with health records collected during the and after Covid-19 lockdown measures (Table 3). While sensitivity remained similar, with both models able to predict approximately 62% of next missed visits, PPV decreased by 2% translating to 17.5% next visits labelled as missed to be truly missed (Table 3). Model performance was not improved with the addition of a singular categorical predictor describing previous visits archetypes (Table 3). In comparison to the full model containing information on historical visits attendance, baseline demographics and clinical features, a model using only previous visit archetypes was able to correctly predict almost half of next missed visits, with a small decrease in precision (PPV of 16.5%).”

We had edited and clarified the opening paragraph of the discussion (lines 415 – 422):

“ART patient retention is a priority for achieving epidemic control in the South African HIV epidemic. To design effective intervention strategies, there is a need for more precise descriptions of the longitudinal changes in the risk of LTFU as well as the characteristics of those who disengage from treatment [26]. We have previously reported that a machine learning model informed by historical visit attendance, baseline demographics and clinical risk factors is able to predict up to two-thirds of next missed ART clinic visits [17]. Here, we demonstrate that historical visit attendance alone is able to predict up to half of next missed visits and it is predictive of IIT using machine learning and traditional statistical methods.”

Withing the discussion We have expanded on the interpretation and usage of these findings on lines 445 – 467:

“While the inclusion of demographic and clinical features improves model performance, we have demonstrated with both machine learning and traditional statistical methods, that historical visit attendance is a strong predictor of IIT. Furthermore, lateness and repeat patterns of lateness can predict IIT, irrespective of current age, age at ART initiation, sex and baseline CD4. The usage of machine learning models such as this are limited to clinical settings where infrastructure exist to extract EMR data and run computations in real-time. In settings where this is not possible, patient archetypes based on historical visits attendance may be used to triage patient retention interventions.”

And we have edited and clarified the closing paragraph of the discussion (lines 415 – 422):

“In this study, we describe baseline and time varying predictors of ART treatment in South African PLHIV. Longitudinal trajectories of ART visit attendance demonstrate that patients transition in and out of treatment indicating that patient retention in South Africa is likely underestimated. Historical visits attendance is predictive of future interruptions in treatment and can be used to identify those at risk of disengaging from HIV care in the absence of other behavioural or observable risk factors.”
